# Contributions of Neuropsychology to the Study of Ancient Literature

**DOI:** 10.3389/fpsyg.2018.01092

**Published:** 2018-06-28

**Authors:** Franco Fabbro, Anastasia Fabbro, Cristiano Crescentini

**Affiliations:** ^1^Department of Medicine, University of Udine, Udine, Italy; ^2^Perceptual Robotics (PERCRO) Laboratory, Scuola Superiore Sant’Anna, Pisa, Italy; ^3^Department of Psychology, University of Rome “La Sapienza”, Rome, Italy; ^4^Department of Languages and Literatures, Communication, Education and Society, University of Udine, Udine, Italy

**Keywords:** neuropsychology, hermeneutics, literature, antiquity, soul, meditation, dreams

## Abstract

The present work introduces the neuropsychological paradigm as a new approach to studying ancient literature. In the first part of the article, an epistemological framework for the proper use of neuropsychology in relation to ancient literature is presented. The article then discusses neuropsychological methods of studying different human experiences and dimensions already addressed by ancient literatures. The experiences of human encounters with gods among ancient cultures are first considered, through the contributions of Julian Jaynes and Eric R. Dodds. The concepts of right and left in the Bible, and that of soul are then discussed. Ecstatic experience in Paul of Tarsus is also presented, with a particular focus on glossolalia. Neuroscientific differences between mindful and unitive meditative practices are then described referring to ancient Buddhist literature, and finally a brief description of dreams in ancient Greek literature is proposed. Neuropsychology variously enables a more profound understanding of themes characterizing human experiences that ancient literature has already explored; these investigations prove that the collaboration of neuroscience and humanistic studies can return fruitful and interesting results.

## Introduction

The scientific experimental method, developed in the West by great scientists and philosophers like Galileo Galilei, René Descartes and Isaac Newton, addressed the study of objects and processes situated in the external environment, in particular the laws that govern the celestial bodies. The study of human anatomy and physiology, developed in the second half of the 19th century by anthropologists and physicians like Pier Paul Broca and Carl Wernicke, contributed to the beginning of the scientific investigation of human cognitive functions, such as language, memory and consciousness (Hurrington, 1989; Finger, 2001). The progressive development of neuroscience and, in particular, neuropsychology provided a great amount of information about peculiar aspects of human cognition, such as the cerebral representation of languages in bilinguals (Fabbro, 1999; Paradis, 2004), the organization in the brain of literacy and numeracy (Dehaene, 1997, 2009) and of emotions (LeDoux, 2016).

One of the most engaging scientific challenges is the possibility to use these knowledges to scientifically investigate the cultural products of the past. Similar approaches have already been adopted, for example in the study of the cerebral networks underpinning the construction of lithic instruments (Stout and Chaminade, 2007; Morgan et al., 2015), or in the neuropsychological interpretation of the prehistoric cave paintings (Lewis-Williams and Clottes, 1998).

Another interesting field in which to introduce a neuropsychological framework concerns the analysis of some aspects of ancient texts, like the Iliad and the Odyssey, the Hebrew Bible, the New Testament, or the ancient Buddhist literature. In this regard, the main aspects of historical-critical methods applied to ancient literature should be taken into account (Gombrich, 2009; Fabbro, 2014a). However, neuropsychological analyses of ancient literary texts shall not constitute in any way an exegetical study of religious texts. Indeed, neuropsychology is not interested in providing any religious interpretation. The neuropsychological approach maintains a neutral attitude in regard of the contents of ancient literary and religious texts. This does not exclude that exegetes interested in neuropsychological analyses of ancient texts can use these information in their studies.

Following these epistemological considerations, 20 years ago, Fabbro (1994, 1995) studied the Hebrew Bible and New Testament from a neuropsychological perspective. In the following sections, we present a critical review of studies that have applied a neuropsychological approach to ancient literature.

Neuropsychology is the field of neuroscience that describes cognitive functions in relation to brain structures. It is distinct from general and clinical psychology, as it concerns the cerebral organization of the psychic function rather than the interpretation of psychic contents: it describes the mode of operation of cognitive and affective systems in relation to different morphological structures of the human brain (Kolb and Whishaw, 2015; LeDoux, 2016). For this reason, neuropsychology can effectively shed light on several aspects of human cognition such as language and narration, memory, dreams, visions and religious experiences in general (Hobson, 1988; Chalupa and Werner, 2003; Tulving and Craik, 2005; Stemmer and Whitaker, 2008; Zeki, 2009; Fabbro, 2010; Gottschall, 2013; van Elk and Aleman, 2017). All these themes are broadly represented in ancient literature.

## Some Considerations Regarding the Scientific Method

In the first half of the last century, the philosopher Karl Popper (1902–1994) developed a series of reflections in order to distinguish scientific and metaphysical statements. According to Popper, scientists begin their studies by formulating a series of theories about nature and then test these theories with experimental data. In Popper’s opinion, experiments should try to confute rather than prove a theory. From this philosophical perspective, a single experiment is sufficient to disprove a theory. Therefore, a theory merits its scientific *status* only if it can be falsified (Popper, 1959). Nowadays, it is unanimously considered that a theory has to be falsifiable before it can be deemed scientific (Smolin, 2006, 2013). Scientific theories are often seen as ‘visions of the world’ obtained through a process of problem solving; in this respect, the aim of science is not to find scientifically true theories but the best theory amongst those available. According to Thomas Kuhn (1922–1996), science is comprised of different scientific paradigms that follow one another (for example, the Ptolemaic paradigm was superseded by the Copernican paradigm) when revolutions in scientific knowledge occur (Kuhn, 1962). Carl G. Hempel (1905–1997) supposed that scientific theories are models based on how we think the world works (Hempel, 1966). These models exist in a theoretical rather than empirical world until that which we envision becomes consistent with what we see. When theories are no longer adequate, we need to improve or turn them into better models; science learns from its mistakes and only effective theories survive.

The Austrian philosopher Paul Feyerabend (1924–1994) proposed a critical approach to the scientific method (Feyerabend, 1975). In his opinion, two opposite tendencies inspire scientific knowledge: the dogmatic and the pluralist attitude (Feyerabend, 1987). The majority of scientists employ the ‘dogmatic attitude,’ which is often unconscious. Its origins can be found in absolutism’s visions of the world developed in the ancient Near East (Needham, 1954). An obsession for unity and condemnation of variety constitutes the essence of this dogmatic vision. The ‘pluralistic attitude,’ also of ancient origin, has been attributed to the encounter of Sumerian, Assyrian, Babylonian and Greek cultures and languages in the Near East. Indeed, the first systems that systematized knowledge were lists of Sumerian words denominating objects in different languages for commercial transactions. Therefore, these rudimentary forms of knowledge arose in a multicultural and plurilinguistic context with a practical purpose. Gradually, stories with a temporal frame were added to these lists that were both simple, such as medical stories from the *Corpus Hippocraticum*, and complex, as in epic poems, tragedies and stories concerning the origins of the world.

## Levels of Analysis in Scientific Research

The different ‘sectors’ that seemingly divide human knowledge could be compared to the departments within a University: mathematics, physics and chemistry, biological sciences (biology, agriculture, medicine and psychology), humanities (philosophy, languages, religious studies, art and music) and social sciences (anthropology, sociology, political science and economics) (Gray, 2010). One philosophical issue, which has generated many discussions, concerns the type of relationships that exists between the different disciplines. In Western culture, we identify two opposite attitudes: on the one hand, there is a search for hierarchical unity; on the other, the recognition of the benefits of a pluralist vision (Craver, 2005; Dyson, 2008; Ayala and Arp, 2010).

In Michael Polanyi’s (1891–1976) famous article, *Life’s Irreducible Structure* (1968), the Hungarian chemist identified certain factors that explain the inability to reduce knowledge to an inferior level. In the author’s opinion, both organisms and machines present two distinct levels: a superior level, which concerns the subject, and an inferior level, which concerns its physical and chemical elements. Polanyi’s key concept suggests that the superior level imposes boundary conditions on the inferior level, even though these boundaries do not violate any physical or chemical law. In other words, both organisms and machines present, at the superior level, a higher thermodynamic order in comparison with the surrounding environment. If they are decomposed and analyzed at the inferior level, the information contained at the superior level is lost in the decomposition. According to this perspective, although cognitive processes are generated and sustained by physical bases, if we were to reduce the psychological and cognitive processes to neurophysiological processes, we would lose lots of information (Polanyi, 1974).

In order to explain how each level operates under the control of the superior rather than inferior level, Polanyi employed the metaphor of various levels in scripture proposed by Democritus and Aristotle (*De Generatione* A1 315b 6; 722a 28ss). A story or ‘text’ is composed of sentences which, in turn, contain words that are formed of letters. Each superior level imposes certain boundary conditions on its inferior levels. The decomposition of a text at the inferior level can be useful. However, while a theory can explain how the letters of the alphabet are arranged in a story (level of the letters), there is no theory or mathematical formula that can tell us anything about the interpretations of that story. The boundaries of superior levels — that is to say the way letters are ordered in words, words in sentences and sentences in a text help to explain the meanings or the ‘semantic relationships,’ in Polanyi’s words, of a story (Polanyi, 1968).

We can conclude that each level of scientific study represents an interesting perspective from which to investigate reality; each level can be studied ‘independently’ of the other levels of analysis (Deutsch, 1997; Feynman, 1998; Kauffman, 2008). The aim of this epistemological introduction underlines how, despite the undeniable connections between different fields of human knowledge, each scientific and humanistic discipline may also be considered autonomous (Feyerabend, 1987; Fabbro, 2014b). Since human knowledge is a highly complex system, it is useful that different disciplines and research methods converge to the comprehension of some key aspects of human experience. A pluralistic approach to human research has become typical in the neuroscientific field, where data provided by clinical, electrophysiological, brain stimulation (e.g., transcranial magnetic stimulation, TMS), and neuroimaging studies are usually considered together in order to detect possible convergences and divergences (Urgesi et al., 2010; Crescentini et al., 2014, 2015). In principle, a clinical neuropsychologist might not be interested in electrophysiological data, and in the same way scholars of papyrology might not be interested in methods of radiocarbon dating. Nevertheless, on both a practical and theoretical level, an interaction between the different fields and subfields of knowledge should be promoted. This approach would benefit research in terms of solidity (due to the convergence of findings) and promptness (many ideas and solutions may be adopted from other disciplines). For these reasons, we consider it useful to emphasize that every field of knowledge can benefit from the influence of other disciplines (see for example Oatley, 1992, 2012).

## The Neuropsychological Organization of the Mind in Antiquity

American psychologist Julian Jaynes (1920–1997) was the first to apply the neuropsychological paradigm to studies of ancient literary texts in his famous book, *The Origin of Consciousness in the Breakdown of the Bicameral Mind* (Jaynes, 1977). According to Jaynes, the encounters between men and divinities, which have been described in the *Iliad* and other ancient texts, should be considered plausible events, as these type of experiences were well known to the ancients. In the author’s opinion, such events are not the result of fantasies or literary genres typical to antiquity but represent a peculiar trait of the archaic mind; in the archaic era, auditory hallucinations were usual, and guided human choices and behavior. Gods’ interventions described in the *Iliad* were in fact the voices and instructions that men heard in visual-auditory hallucinations. According to Jaynes, the archaic mind was a ‘bicameral mind’ divided in two parts; in particular conditions, the right cerebral hemisphere generated visual and verbal hallucinations that the left “verbal” hemisphere interpreted as gods’ messages or orders. Thus, the two hemispheres worked as two independent units: man (the left cerebral hemisphere) and god (the right cerebral hemisphere). A similar approach, based on philological and anthropological reflection, had already been developed by Eric R. Dodds (1893–1979) in his famous text, *The Greeks and the Irrational* (Dodds, 1951). Jaynes’s theory has generated great interest. Although the central aspect of his theory — namely the existence of the bicameral mind in antiquity — has been questioned (Cavanna et al., 2007), two other aspects of Jaynes’ work are, in our opinion, highly significant. The first concerns an attempt to apply the neuropsychological paradigm to the study of ancient literature. The second invites researchers to investigate the complexity of the ancient world from a more complex perspective than the typical philosophical viewpoint of the Western culture. Importantly, the latter dominant perspective, which emerged after the Enlightenment, tends to underestimate a fundamental aspect of all ancient traditions: the search for altered states of consciousness, such as enthusiasm, ecstasy and dream, *as a way* to search meaning in life (Eliade, 1951; Couliano, 1984).

Both Dodds and Jaynes attempted to comprehend the frequent representation of these events in ancient literature from an anthropological and neuropsychological perspective. After all, some scholars studying the ancient world had already underlined the possibility that descriptions concerning encounters with the divinities in ancient Greek literature and the Hebrew Bible could be related to real experiences (Rohde, 1907; Gunkel, 1917). More recent research suggests that a psychological and neuropsychological framework for interpreting ancient texts could be very revealing. For example, it is thought that Pythia’s trance at the Oracle of Delphi was caused by the inhalation of a gas with psychoactive properties emanating from a crevice within the temple of Apollo. The ceremony has been described in detail by Strabo (64 BCE - 25 CE) and by Plutarch (47–120 CE) (Plutarch, 1936, 42.50–51). Some studies suggest that the inhalation contained ethylene gas, a volatile substance able to induce states of *trance* that can eventually lead to a complete loss of consciousness (Hale et al., 2003).

## The Neuropsychology of ‘Right’ and ‘Left’ in the Bible

After reading Jaynes’ work, one of us decided to apply the neuropsychological paradigm to concepts of ‘right’ and ‘left’ in the Bible (Fabbro, 1994, 1995). The functional differences between the right and left hand correlate to the two cerebral hemispheres’ distinct specializations (McManus, 2004). Robert Hertz (1881–1915) analyzed the differences between right and left from an anthropological point of view (Hertz, 1909). He observed that, in a variety of human cultures, religious organization is based on fundamental dichotomies that oppose the sacred (related to the right) and the profane (related to the left). In his opinion, these asymmetries were not determined by cultural factors but by biological bonds related to distinctions between the two hemispheres (Chasteen et al., 2010).

Biblical texts have been analyzed in their original languages using historical and critical research methods that identify all occurrences of terms meaning ‘right’ and ‘left’ (Fabbro, 1994, 1995). In the Jewish Bible, 177 verses contain either of the two terms, while New Testament verses contain 49 references. For each verse, we proposed an anthropological classification and for complex verses (e.g., Judges 20:14–16; I Samuel 11:1–2; Proverbs 3:16; Psalms 10:1; 137:5–6; Jonah 4:11), which are highly contentious in exegetics, some possible neuropsychological interpretations was given in addition to common readings. The descriptive statistical analysis of these verses is interesting: in the Hebrew Bible, while 105 verses solely concern the right (82.6%), only 22 verses just concern the left (17.3%) with 72 verses concerning both right and left; meanwhile, in the New Testament, 44 verses concern the right (91.6%) and only 4 verses the left (8.3%). Both in the Hebrew Bible and New Testament, the right is mentioned more often than the left. This could be an index of the aversion and fear that cultures and religions, including Jewish and Christian religions, may have had toward the left; generally, people tend not to mention that which frightens them. Consistent with this consideration, Tan (1998) also found that the right hand is mentioned more frequently than the left in the Koran: while the right hand is recorded in 23 sentences, the left occurs only 11 times.

In the Hebrew Bible, the number of references to the left is twice that of the New Testament (17% versus 8%) which likely reflects the amount of archaic influences in the former, whose authors and editors, whilst conveying the usual fear toward the left, were more inclined to align themselves with its unpredictability.

In the Hebrew Bible, the books that contain more references to the right and / or the left are: the Psalms (39 verses = 22%), Kings (15 = 8.4%), Leviticus (13 = 7.3%), Chronicles (12 = 6.7%), Ezekiel (11 = 6.2%), Samuel (10 = 5.6%) and Isaiah (10 = 5.6%). In the New Testament, the books that contain more references to the right and / or the left are: Matthew (12 = 21.4%), Revelation (9 = 16%), Acts (8 = 14.2%), Mark (7 = 12.5%), Luke (6 = 10.7%) and Hebrews (5 = 8.9%). These descriptive statistical analyses help us comprehend certain aspects connected to the approaches of the biblical texts’ authors. For example, all the letters of Paul from the New Testament — excluding the Letter to the Hebrews with its five verses that amount to 8.9% of all New Testament verses — contain very few references to the right and left. It is improbable that an author such as Paul, who almost never employed the terms right and left, would have used them so frequently in only one of his books. Based on this simple analysis, we can hypothesize that the Letter to the Hebrews was not written by Paul but attributed to him at a later stage. In keeping with this analysis, many modern studies do not attribute the Letter to the Hebrews to Paul the Apostle (Hurst, 1989). Additionally, two texts that are traditionally attributed to John the Apostle — the Gospel of John and the Revelation — include a different number of references to the left and the right: while in the Gospel, there are only two citations (3.5%) that refer to the right, in the Revelation right and left terms are reported nine times (16%). In line with these considerations, other linguistic, stylistic and exegetic parameters allow us to conclude that the two aforementioned texts were composed by two different authors, who probably belonged to the same religious tradition (Fabris, 2003).

Many passages from the Hebrew Bible and New Testament can be better interpreted in light of knowledge provided by neuropsychology. For example, the common preference for referring to the right hand in the Bible is frequently associated with positive characteristics, such as strength, power, honor, safety and justice (Fabbro, 1994, 1995). Consider, for example, Deuteronomy 33:2: “And he said: The Lord came from Sinai, and rose from Seir unto them; He shined forth from mount Paran, and He came from the myriads holy, at His right hand was a fiery law unto them.” The most significant part of this passage, *mîmînô ’ ēšhdāt lāmô*, has been translated by rabbis as “From His right, for them, the fire of the Law.” The rabbi Rashi argues that God wrote the Law, the *Torah*, with his right hand. According to the latter verses of Deuteronomy, God wrote the Ten Commandments on tablets of stone with fire that came from his right hand. Based on this important passage, other texts concerning the polarity of right and left from the Hebrew Bible can be interpreted similarly. Therefore, “a wise man’s understanding inclines toward his right, but a fool’s understanding toward his left” (Ecclesiastes 10:2) suggests that the wise man who directs his heart to the *Torah*, to the right of God, is rewarded with a long life (Proverbs 3:16). As in the Old Testament, the New Testament presents a similar dichotomy between right and left. For example, in the Gospel of Matthew (25:31–34), when the “Son of man comes in his glory, in separating the nations, “he will put the sheep on his right and the goats on his left”: the former will live an “eternal life” and the latter “will go away to eternal punishment.”

From a neuropsychological perspective, Psalms 137, verses 5–6, are collectively another important passage, which were probably composed during the Babylonian exile from 540 to 515 BCE: “If I forget thee, O Jerusalem, let my right hand wither; let my tongue cleave to the roof of my mouth, if I do not remember thee.” In these verses, the author infers an association between the paralysis of the right hand and language impairment: both symptoms, in effect, are caused by a lesion in the left cerebral hemisphere, which controls the motility of the right side of the body and language. The meaning of the expression “let my tongue cleave to the roof of my mouth” is clarified in Ezekiel 3:26, where the inability to speak is referred to: “I will make thy tongue cling to thy palate and thou shalt be dumb.” In both texts, the authors are probably describing an acquired language disorder (aphasia), which compromises the inability to speak and impedes the tongue’s voluntary movements. The psalmist impresses upon those who forget Jerusalem one of the worst possible tragedies: the loss of the dominant hand’s use and that of language, two abilities that are associated with writing, reading and praying, without which a practitioner would no longer be able to participate in religious rituals and, therefore, be considered impure (Fabbro, 1994, 1995). The neuropsychological analysis of the concepts of ‘right’ and ‘left’ in the Hebrew Bible and the New Testament confirms the tendency to connect the right side to the law, order and justice, and the left side to the unpredictable, chaos and divination. Since this dichotomy is present in many different human cultures (Hertz, 1909; Needham, 1973), it has been connected to the asymmetry of the cerebral functions (Hertz, 1909; McManus, 2004). This datum highlights the utility of a neuropsychological interpretation of the cultural dichotomy of right *vs* left.

## The Out-Of-Body Experience and the Concept of Soul

The concept of ‘soul’ (*anima* in Latin) is widespread in many cultures and religions. It derives from the Greek term *anemos* (meaning ‘the wind’) and is connected to the concept of spirit (in Latin, *spiritus*, meaning ‘breath of life’) (Benveniste, 1969). In common language, soul refers to something airy, able to move and fly away from the body. The shaman is considered a specialist in ecstasy and leaving the body, a condition which allows him to magically ‘fly’ from the human world to that of otherworldly powers (Eliade, 1951). In many cultures, such as that of Ancient Egypt, the soul is represented by a bird that leaves the body when an individual dies (van der Leeuw, 1956). Among the Ancient Greeks, *psychē* is an image (*ēidōlon*) analogous to that which an individual can see in the mirror or in a dream. It does not coincide with the body nor mind, even though it is responsible for their vitality. The *psychē* can leave the body during dreams, ecstasy and, above all, in death (Rohde, 1907). It is commonly believed that the concept of soul is at the basis of dualistic philosophical concepts (Metzinger, 2009).

Recently, scholars have proposed a hypothesis that can explain the ubiquitous origin of the concept of soul in many cultures linking it to the well-known Out-of-Body Experience (OBE) phenomena. OBEs are conditions in which an individual simultaneously presents two models of the self: a self-as-object (perceived in the third person, often laid down in bed) and a self-as-subject (perceived in the first person), which is generally characterized by a light, floating effect. Importantly, individuals tend to identify themselves with the fluctuating self (phantasmal entity) as opposed to the object-self (body) during these experiences. Studies have documented that OBEs are quite frequent phenomena among the general population (10%), very frequent in subjects with schizophrenia (40%), even more frequent in individuals near death (60%) and common in patients with focal epilepsy due to a lesion or a dysfunction of the temporoparietal junction of the right hemisphere, a multisensory brain region integrating inputs from different sensory modalities (Metzinger, 2009; van Elk and Aleman, 2017). Furthermore, it has been underlined that the electrical stimulation of the right temporoparietal junction, during interventions of neurosurgery with conscious patients, can produce repetitive OBEs (Blanke et al., 2002, 2004). With reference to anthropological, philosophical and neuropsychological data, Thomas Metzinger hypothesizes that the concepts of soul and spirit in many cultures originates from OBE manifestations and narrations (Metzinger, 2005).

Out-of-Body Experiences are categorized under autoscopic phenomena, which are conditions where a subject is able to see his body from the outside. Besides OBEs, the main forms of autoscopy are autoscopic hallucinations, heautoscopies and the sensation of presences. In autoscopic hallucinations, the individual sees his body from the outside without identifying with it. A patient experiencing heautoscopy phenomena does not always know what his body is (Blanke et al., 2004; Metzinger, 2009). Finally, in the sensation of presence, the individual has the sense of a second body next to him, which is felt but not seen. These conditions can be associated to specific lesions or functional alterations of the temporoparietal lobes (Irwin, 2003). However, other non-ordinary states of consciousness achieved through particular techniques (ascetic or sensorial and dream deprivation) and epilepsy can also determine autoscopic phenomena. The renowned Russian novelist Fëdor Michajlovič Dostoevskij (1821–1981) described many autoscopy experiences, namely in his novel, *The Double*, published in 1846 (Fabbro, 2003). In some recent studies on Jewish mysticism, specific techniques and teachings of the Cabala have also been associated with certain autoscopic phenomena (Arzy et al., 2005; Idel, 2008). Many reports of phenomena associated to ecstasy and modifications of the self in ancient literature (e.g., rise to the Heaven, feelings of presences, OBE experiences), can thus be analyzed not only from a philological, hermeneutic and symbolic point of view, but also from a neuroscientific perspective (Ananthaswamy, 2015). Neuroscientific studies concerning OBE, autoscopy and near-death experiences, can shed light on whether such descriptions in ancient literary texts refer to real experiences or more likely to works of fiction or literary genres. Furthermore, neuroscientific studies can help us to foster our understanding of the origin of some universal and complex concepts like that of soul.

## Neurolinguistic Analysis of Glossolalia in Paul of Tarsus

The teachings of the apostle Paul of Tarsus (c. 5 – c. 67 ACE) provide an interesting example of the relation between biblical studies and neuropsychology. Very interesting in Paul’s preaching is the phenomenon of glossolalia. The term ‘glossolalia’ is derived from the Greek words *glossa*, ‘tongue,’ and *laléo*, ‘to speak,’ and is also known as ‘speaking in tongues.’ It refers to the experience to speak in unknown languages, generally in religious contexts (Cartledge, 2002). The complex phenomenon of glossolalia has been studied from different perspectives that consider its possible connection with a variety of psychological, physiological and socio-cultural factors such as trance, psychopathology, hypnotic susceptibility and anticipated / usual religious acts in religious prayer groups (Samarin, 1973; Spanos and Hewitt, 1979). More recently, Grady and Loewenthal (1997) distinguished two main forms of glossolalia: the first type, involving individuals who are aware and speak calmly, occurs frequently and commonly in private and non-religious settings; the second type, involving individuals who are excited and unaware whilst experiencing an altered state of consciousness that combines vocal utterances, singing and ecstatic bodily experiences, occurs occasionally in public and religious settings. The second type has been studied by Newberg et al. (2006) in a functional neuroimaging investigation using SPECT on five practitioners of glossolalia. In this study, a significant decrease in the prefrontal cortices during glossolalia was consistent with the perceived loss of an individual’s intentional control over their vocalizations. Moreover, glossolalia was associated with decreased activation of the temporal pole. In this respect, it is interesting to note other studies that have discussed the relationship between glossolalia and temporal lobe activity: in particular, Persinger (1984) described the case of a young woman who displayed temporal lobe spikes during episodes of glossolalia, a finding similar to that documented more recently by Reeves et al. (2014).

Paul of Tarsus refers to glossolalia within the first epistle to Corinthians, verses 14:1–40 (Fabbro, 1998; Shantz, 2008, 2009). The motivation that may have induced Paul to compose this text can be attributed to the fact that the charisma of prophecy was disregarded in favor of the glossolalic experience in the Corinthian ecclesiastical assembly (Barbaglio, 1996). In order to promote growth of the assembly (v.4), Paul proposed a hierarchy of the charismas. The most important of them is the revelation (v.31), then the prophecy (v.29), while the glossolalia occupies third position. In particular, glossolalia can only be practiced if an interpreter explains its content to the community immediately after the glossolalic expression (v.29) or the person who experiences the glossolalia translates what he has said to the assembly. If there is not interpreter, Paul’s writing suggests that the glossolalic is silent (v.28). However, Paul was not against uninterpreted glossolalia in principle: contrary to the opinion expressed by the author of the Acts in the narration of Pentecost, Acts 2:1–13, he did not think that it was a foreign language; he believed that glossolalia was a prayer expressed in ecstasy not addressed to humans but to God (vv.1–4).

Neurolinguistic analyses of glossolalia seem to confirm Paul’s opinion: no human language presents such a high percentage of repetitions (echoisms) as the glossolalic production (Lebrun and Fabbro, 2002). Paul did not support the glossolalic expression without interpretation, because, in his opinion, it could cause the ‘upset’ (*acatastasia*) of community rules (v.33). In the first Epistle to the Corinthians, Paul did not want to simply prevent the ecclesiastic assembly needlessly hearing verbal expressions without meaning; it is possible that he wanted to impede the collective ecstasy and sexual disinhibition that glossolalic expressions might induce during the assembly. This suggests that Paul knew the practice of glossolalia could have effects that were probably due to emotional contagion phenomena (Panksepp, 2009; Prochazkova and Kret, 2017). In fact, in v.40, Paul invites the followers of Corinth to ‘*decently*’ (*euschemonōs*) take part in the Ecclesiastical assembly and uses this term in other contexts (1 Corinthians 7:35; Romans 13:13) with reference to sexual behavior. Interestingly, Paul consented to glossolalic expressions during Ecclesiastical assemblies, as long as people spoke one at a time and that their expressions were immediately interpreted in a comprehensible language (v.27). Furthermore, according to Paul, glossolalic or prophetic expressions could not be more than three per meeting (vv.27–29). All these prescriptions were probably aimed at limiting emotional contagion and collective ecstasy phenomena among those at the assembly.

Many authors and biblical scholars have claimed that ecstasy was a fundamental dimension in Paul’s life (Shantz, 2009). The analysis of Paul’s ecstasy experiences in the light of neuropsychological and religious studies, led Fabbro (1998) and Shantz (2009) to conclude that the ecstasy described by Paul in his letters refer to authentic personal experiences. Probably, Paul was able to achieve the knowledge and the intuitions that were at the basis of his predication (particularly in the first letter to Corinthians) thanks to his ecstatic experiences.

## Contributions of Neuroscience to the Study of Ancient Buddhist Literature

Another example of the application of the neuropsychological paradigm to the study of ancient literature concerns historical and critical research investigating the origins of Buddhism. This religious tradition, introduced in the 5th century BCE by Indian prince Siddhartha Gautama, known as the Buddha, considers meditation at the core of this devotional practice. Indeed, the last two steps of the Buddhist Path to liberation (known as the Noble Eightfold Path) concern two different meditative practices (Gombrich, 2009): the seventh step, *sammā sati*, which refers to mindfulness meditation, and the eighth step, *sammā samādhi*, concerning unitive meditation. Researchers interested in the origin of Buddhism have examined the Buddha’s unique contribution to meditation after that many meditative practices had already been established by the Hindu tradition.

According to tradition, before achieving spiritual realization, Prince Siddhartha Gautama was trained in Hindu meditative techniques by two Brahmins: Alara Kalama and Udakka Ramaputta (Gombrich, 2009). From these teachers, the Buddha learned techniques to achieve the *samādhi*, a mental condition that he considered necessary but not sufficient for complete spiritual realization. In the opinion of Alexander Wynne, an historian of the origins of Buddhism, the Buddha introduced mindful awareness (of breath, the body and mental states) to meditative practices (Wynne, 2007). According to Buddha, mindful awareness (*sammā sati*) is a fundamental prerequisite for reaching the eighth and final step of the spiritual realization path (*sammā samādhi*). In Wynne’s opinion, meditations inspired to Buddhist tradition allow to reach two cognitive states: mindful awareness and the unitive state, while practitioners of Hindu meditation are restricted to states of *samādhi*.

As this is an historical-philological hypothesis that can be verified, we recently performed meta-analysis of all neuroimaging studies including experiments that used groups of subjects practicing Buddhism-inspired meditation (16 studies with 263 subjects) and Hinduism-inspired meditation (8 studies with 66 subjects) (Tomasino et al., 2014). In the meta-analysis, the studies were classified as related to Buddhism- *vs* Hinduism-inspired meditation on the basis of the religious tradition they referred to; more recently, other classifications of meditation practices have been proposed based primarily on the underpinned psychological and cognitive mechanisms (Dahl et al., 2015). The results of the meta-analysis by Tomasino et al. (2014) were elaborated with a specific statistical technique (‘activation likelihood estimation meta-analysis’) and underlined a critical role of the parietal lobe (temporoparietal junction) in Hinduism-inspired meditation. As previously mentioned, this region is associated with unitive experiences and OBE phenomena. Moreover, the results showed a key activation of frontal and prefrontal regions in practitioners of Buddhism-inspired meditation, which could be connected to executive functions and voluntary attention required during mindful awareness (*sammā sati*, Nyanatiloka, 2001; Tomasino et al., 2014; see also Tomasino et al., 2013). This neuroscientific research appears to confirm the historical-philological hypothesis proposed by Wynne, aligning the Buddha’s meditation advances with voluntary attention practiced through mindful awareness (Fabbro and Crescentini, 2014). In conclusion, neuroscientific studies (e.g., meta-analysis of neuroimaging studies) can support the validity of philological hypotheses. In the mentioned example, Wynne’s proposal that the main innovative contribution of the Buddha in terms of meditation concerned the development of right mindfulness, appeared to be confirmed by functional imaging results showing a preferential activation of frontal lobe regions in meditation practices inspired by the Buddhist religious tradition.

## Neuropsychological Analysis of Dreams in Ancient Greek Literature

Neuropsychology’s contribution to the study of ancient literature also concerns dreams. Dream analysis distinguishes between ‘dream form’ and ‘dream content’ (Hobson, 1988): the *form* of dreams is supposed to be universal and neuropsycologically determined; the *content* of a dream is linked to individual experiences and appears to be influenced by culture and gender (Domhoff and Schneider, 2008).

Many characteristics of dream form can be distinguished in the REM phase of sleep. First of all, dreams are characterized by vivid images: sight predominates over other senses (Hobson, 1988). Auditory perceptions are also quite common, while touch, smell and taste sensations are rare (Hobson, 1988; Schredl and Wittmann, 2005; Desseilles et al., 2011). Another characteristic of dreams is ‘bizarreness’ which, according to Hobson (1988), concerns three main aspects: (a) plot, characters, objects and action; (b) thoughts; (c) emotions and feelings. Moreover, Revonsuo and Salmivalli (1995) proposed three kinds of bizarreness: incongruity, vagueness and discontinuity. With regards to awareness within dreams, apart from lucid dreamers, people are not usually consciously aware of being in a dream: they think that characters and events are real, even when bizarre, and accept them as if they were normal. Furthermore, the dreamer does not generally have control over the content of his / her dreams (Desseilles et al., 2011).

Another characteristic of dreams relates to memory. Hobson (1988) observed that during dream the dreamer’s mind seems “hypermnesic,” experiencing amnesia on awakening: it is considered that dreamers forget more than 95% of their dreams. High levels of emotional involvement also characterize dreams: euphoria, anxiety, fear and anger are more frequent dream-state emotions than sadness, guilt and shame (Hobson et al., 2000). Narrative plot is also a dream characteristic (Hobson, 1988). Additionally, the dreamer maintains the ability of ‘mind reading’ during dream and can thus attribute feelings, thoughts and intentions to other characters. Given that all these characteristics have neurobiological bases, we could presume that the dreams of ancients were characterized by these same features.

Greek literature presents distinct examples of dream reports. In particular, The *Sacred Tales* (*Hieroi Logoi*) by Aelius Aristides (117–181 CE) offers a very intriguing collection of dream reports, which are the most ancient, autobiographical ‘diary of dreams’ remaining today (Nicosia, 1988). Aelius Aristides was a famous orator affected by many illnesses who sought guidance from the temple of Asclepius in Pergamum; for several years, the god appeared to Aristides in his dreams prescribing him remedies. When Aristides was older, the god asked him to collect his dream reports, including the prodigious manifestations of Asclepius. Aristides obeyed and wrote the *Sacred Tales.* In this work, which consists of six books, Aristides represented his dreams and their interpretations, adding biographical content, and descriptions of illnesses, therapies and miracles (Behr, 1968).

Many psychological interpretations of the *Sacred Tales* have been proposed and analyses of the latent content of Aristides’ dreams performed (see Stephens, 2012). However, whether Aristides’ reports refer to real dreams or not should precede any attempt at analysis. In recently attempting to answer this question (Fabbro and Fabbro, 2015), we found that not all Aristides’ dream reports are long and descriptive: sometimes he only refers to having dreamed, without relating the dream itself; in other cases, the reports are very short. We analyzed ten longer dream reports (1.10–14; 1.17; 1.22; 1.36–40; 1.46–49; 1.54; 4.48–51; 5.22–24; 5.44–45; 5.57–66), where descriptions consisted of more than 30 Greek words, and they included many of the dream characteristics mentioned above.

All of the ten dreams present visual descriptions and follow a narrative plot. Additionally, Aristides does not seem to be consciously aware of dreaming during these ten accounts. Nine dreams have bizarre content, including elements that are incongruous with reality: “It seemed to me that the majority of the buildings had certain ladders attached, and that I had to go up and down these” (5.65). They sometimes also include discontinuity in time or space: “I went along some path, and next there was a very large vault [...] when to my relief I got through, I appeared to be in the city of Smyrna, in the marketplace” (1.22). Seven dreams present intense emotions from fear and anxiety to happiness and excitement: “I was delighted by the honor and the extent to which I was preferred to the others, and I shouted out, ‘The One’, meaning the God” (4.50). While in three dreams, Aristides displays mind reading abilities, in another he admits to not exactly recalling all his dreams (amnesia). In light of this analysis, we conclude that the ten dreams of Aelius Aristides could be related to authentic dream experiences (Fabbro and Fabbro, 2015; see also Nicosia, 1988, and Harris, 2009).

Interestingly, we have also compared Aristides’ dreams with those contained in the *Iliad*. Three dreams are described in the poem: those of Agamemnon (Book 2), Achilles (Book 23) and Priam (Book 24). Although another dream occurs in the 10th book (Rhesus’ dream), it consists of only a few verses and the dream experience itself is not described. Homeric dreams have been interpreted as autonomous and independent of the dreamer’s mind, as an objective reality (Dodds, 1951; Guidorizzi, 2013). In the so-called ‘objective dream,’ a figure appears to the sleeping person who is passive and reveals a message to him before leaving. The dreamer is always aware of being in a dream, because the dream itself says to him: “you are dreaming.” This type of dream is very different from that which we commonly experience and does not present the psychological characteristics we have previously outlined. From a neuropsychological perspective, these dreams seem to refer to literary fiction rather than real experiences; an analysis of the ‘objective dream’ should consider other factors such as literary context, the formulaic style of Homeric poems and the role of dreams in developing narrative plot.

In conclusion, the neuropsychological analysis of dream descriptions in ancient literature can help us to distinguish those that probably refer to imagination and literary genres (dreams in the *Iliad*) from those that probably refer to real experiences (dreams of Aelius Aristides).

## Conclusion: What Role Can Neuropsychology Have in the Study of Ancient Literature?

In recent decades, many neuroscientists have been interested in humanistic disciplines (Jaynes, 1977; Fabbro, 1994, 1995; Zeki, 2000, 2009; Lehrer, 2007, 2012). Their approaches can be schematically defined by two general categories: (a) those who *explain* humanistic disciplines through neuroscience; (b) those who would like to provide a hermeneutic *service* to the humanities. The first category seems to include the majority of researchers whose studies have been mostly conducted from a reductionist perspective (Jones, 2000; Machamer and Sytsma, 2007). At the basis of this viewpoint is the idea that a science of painting, music and literature can be established through the neurobiological explanation of abstract concepts, such as love, beauty and justice (Ramachandran and Hirstein, 1999; Zeki, 2000, 2009). Although the approach of these studies has been questioned (Miller, 2010; Ayala and Arp, 2010), their contributions can be very interesting. For scholars who study painting or history of art, for example, it can be informative to know more about the neurophysiology of vision or the neuropsychological profiles of patients who are extraordinarily gifted, or including those with autism (Maffei and Fiorentini, 1995; Mazzucchi et al., 1995; Miller et al., 1998). All these studies, however, are only concerned with neuroscience. In the most extreme cases, they imply that a true comprehension of the studied phenomena can only be achieved through neuroscience. This may be misleading, as advances in literature, art and music have continued for centuries, despite almost nothing being known until relatively recently about the brain and its functions.

From a complementary perspective, the *use* of neuroscience and neuropsychology in relation to a hermeneutic approach toward painting, music, literature and religious studies could be a beneficial means to develop a more collaborative approach with humanistic studies in general and ancient literature in particular (Fabbro, 2014b). This approach has already been pursued by Alexander R. Luria (1902–1977) and Roman Jakobson (1896–1982) (Jakobson, 1941; Luria, 1973, 1976; Jakobson and Waugh, 1979). Nowadays, neuroscience, neurolinguistics and neuropsychology maintain a composite name, signifying their innate complexity; Luria and Jakobson were more interested in promoting the interconnections between neurology, linguistics, sociology, art and literature than demonstrating that “we are our brain” (Swaab, 2010). Moreover, the works of American neurologist Oliver Sacks are example of how this collaborative approach between different disciplines can be appreciated by both scientists and the general public (Sacks, 1985, 1995, 2007). Scientific disciplines, such as physics, chemistry and neuroscience, can contribute significantly to humanistic disciplines. Similarly to the methods of radiocarbon dating and statistical studies of literary styles (Schreibman et al., 2004; Levy and Higham, 2005), neuropsychology could foster the understanding of certain key human experiences – visions, dreams and miracles – that are described so profoundly in ancient texts.

Since the final and most important aim of both neuropsychological research and humanistic studies is to investigate and more thoroughly understand the complex dimensions of human nature, it seems necessary for future studies to further promote a close collaboration between scientists and researchers within the humanities (Kidd and Castano, 2013; Willems and Jacobs, 2016). This would not only help to achieve better understanding of the human mind but also a wider and more complete perspective on it.

## Author Contributions

FF developed the theoretical concept and wrote the manuscript. CC and AF contributed viewpoints regarding neuroscience and dreams in Ancient Greek literature and helped write the final manuscript.

## Conflict of Interest Statement

The authors declare that the research was conducted in the absence of any commercial or financial relationships that could be construed as a potential conflict of interest. The reviewer IA and handling Editor declared their shared affiliation.
